# Supplementary material for: Seminoma and Embryonal Carcinoma Footprints Identified by Analysis of Integrated Genome-Wide Epigenetic and Expression Profiles of Germ Cell Cancer Cell Lines
Source: PLoS One. 2014 Jun 2;9(6):e98330. doi: 10.1371/journal.pone.0098330 (PMC4041891; doi:10.1371/journal.pone.0098330)
Supplement: File S1 — ZIP file containing DMRforPairs output for significant regions. Please start from the html files. (ZIP) [file pone.0098330.s008.zip › figures/27531.pdf]

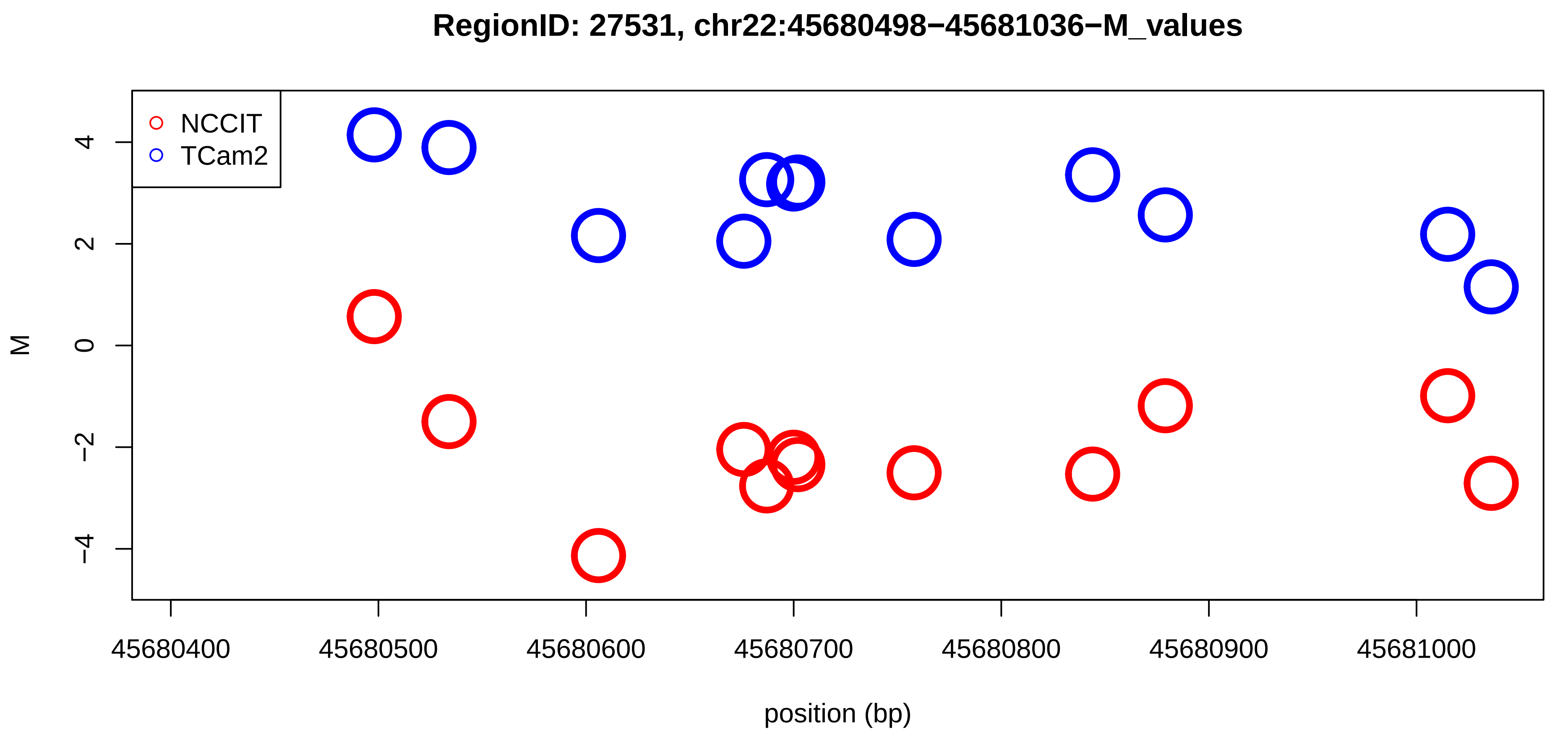
RegionID: 27531, chr22:45680498−45681036−M_values
NCCIT
TCam2
M
4
2
0
−2
−4
45680400
45680500
45680600
45680700
45680800
45680900
45681000
position (bp)

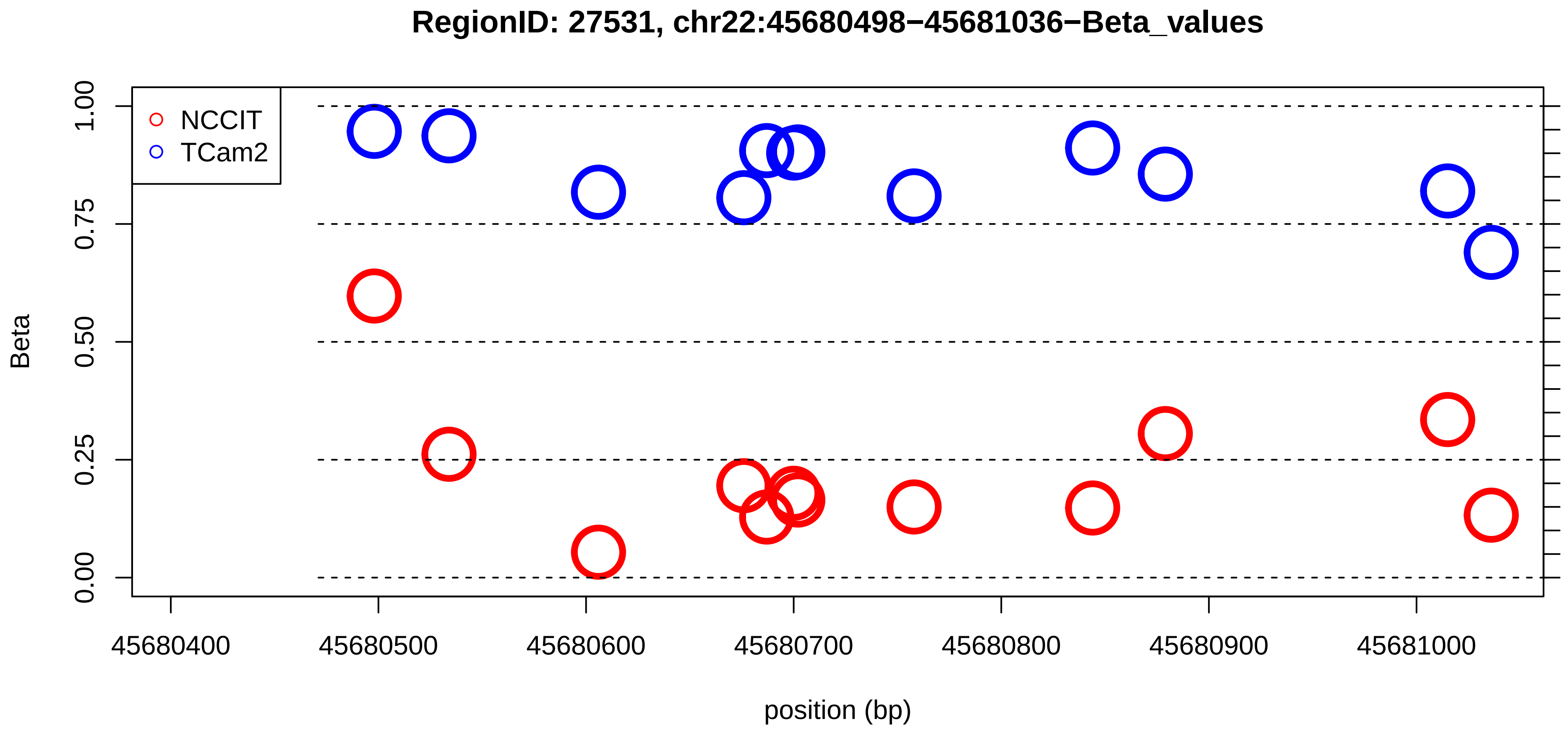
RegionID: 27531, chr22:45680498−45681036−Beta_values
NCCIT
TCam2
Beta
1.00
0.75
0.50
0.25
0.00
45680400
45680500
45680600
45680700
45680800
45680900
45681000
position (bp)

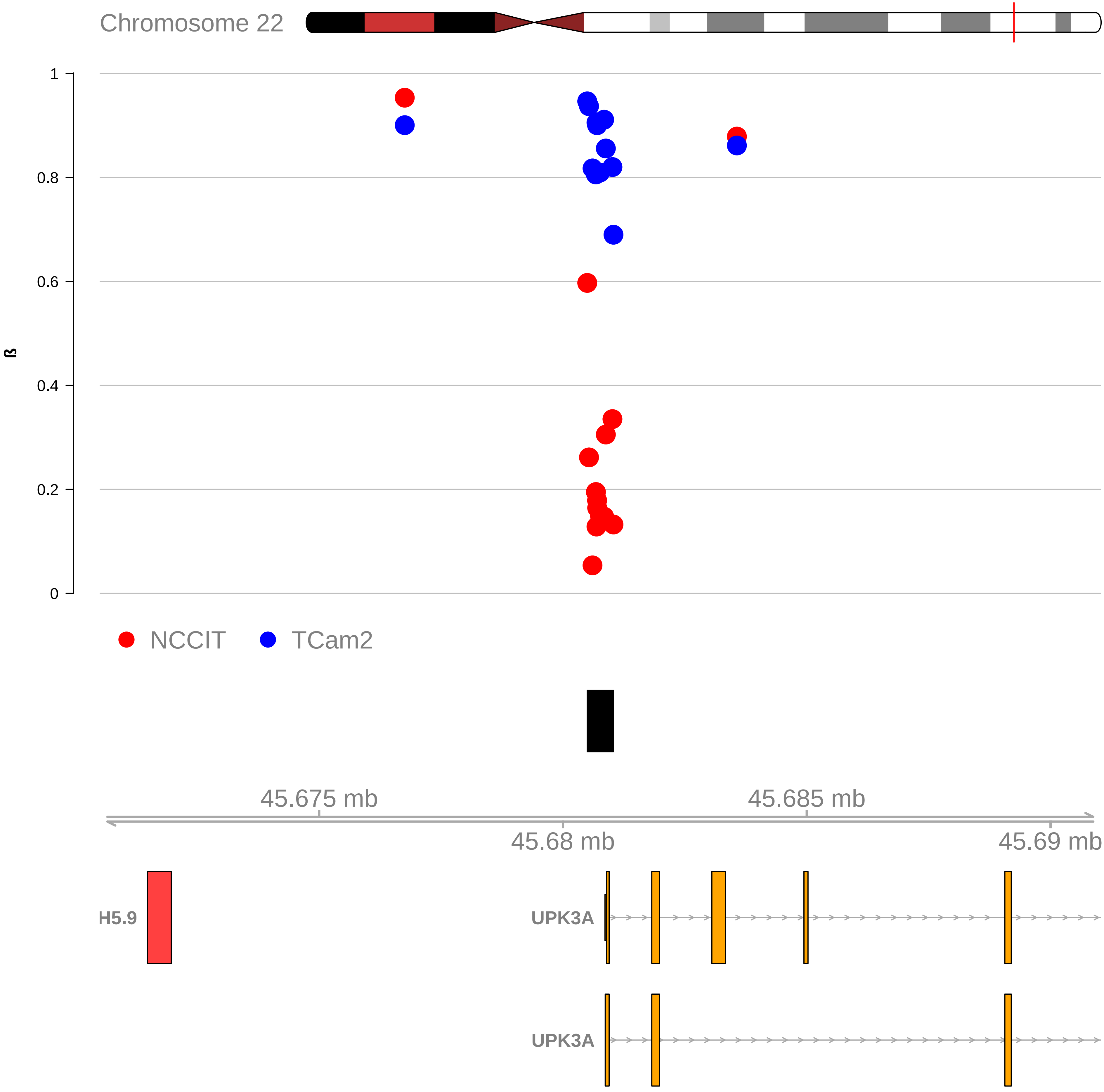
Chromosome 22
1
0.8
0.6
0.4
0.2
0
ß
NCCIT
TCam2
45.675 mb
45.685 mb
45.68 mb
45.69 mb
H5.9
UPK3A
UPK3A
